# 5‐HT6R null mutatrion induces synaptic and cognitive defects

**DOI:** 10.1111/acel.13369

**Published:** 2021-05-07

**Authors:** Zehui Sun, Bingjie Wang, Chen Chen, Chenjian Li, Yan Zhang

**Affiliations:** ^1^ State Key Laboratory of Membrane Biology College of Life Sciences Peking University Beijing China; ^2^ PKU/IDG McGovern Institute for Brain Research Beijing China; ^3^ School of Life Sciences Lanzhou University Lanzhou China

**Keywords:** 5‐HT6R, learning and memory, neuronal excitability, primary cilia

## Abstract

Serotonin 6 receptor (5‐HT6R) is a promising target for a variety of human diseases, such as Alzheimer's disease (AD) and schizophrenia. However, the detailed mechanism underlying 5‐HT6R activity in the central nervous system (CNS) is not fully understood. In the present study, 5‐HT6R null mutant (5‐HT6R^−/−^) mice were found to exhibit cognitive deficiencies and abnormal anxiety levels. 5‐HT6R is considered to be specifically localized on the primary cilia. We found that the loss of 5‐HT6R affected the Sonic Hedgehog signaling pathway in the primary cilia. 5‐HT6R^−/−^ mice showed remarkable alterations in neuronal morphology, including dendrite complexity and axon initial segment morphology. Neurons lacking 5‐HT6R exhibited increased neuronal excitability. Our findings highlight the complexity of 5‐HT6R functions in the primary ciliary and neuronal physiology, supporting the theory that this receptor modulates neuronal morphology and transmission, and contributes to cognitive deficits in a variety of human diseases, such as AD, schizophrenia, and ciliopathies.

## Graphical abstract

Alzheimer’s disease (AD) is associated with changesin synaptic morphology, nervous system dysfunction, and cognitive decline. Lossof 5‐HT6R elevates anxiety and decreases learning and memory ability in mice.Meanwhile, changes in ciliary signaling, dendrites, synaptic plasticity, andneuronal excitability are found in 5‐HT6R mutant mice, suggesting that5‐HT6R has potential therapeutic value in impeding AD progression.

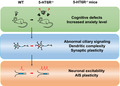

## 1 INTRODUCTION

Serotonin (5‐hydroxytryptamine, 5‐HT) is a biogenic amine most noted for its role as a neurotransmitter and is implicated in pathogenesis of mood disorders (Mohammad‐Zadeh et al., 2008). 5‐HT acts through a variety of 5‐HT receptors (5‐HTRs), which are classified into seven families according to their functions, intracellular pathways, and sequence homology (De Deurwaerdère et al., 2020). Recent studies have indicated that serotonin 6 receptor (5‐HT6R) may be a potential target for cognitive improvement in Alzheimer's disease (AD) (Hu et al., 2017; Khoury et al., 2018; Ramirez, 2013), schizophrenia, and other cognitive or memory deficits. The administration of 5‐HT6R antisense oligonucleotide was reported to induce anxiety‐related impairment (Hamon et al., 1999). Improvements were subsequently observed with the administration of 5‐HT6R antagonists in rat/mice behavior tests for learning and memory abilities (de Bruin & Kruse, 2015; Hu et al., 2017; Woolley et al., 2003). In contrast, 5‐HT6R agonists reduced memory behavior in rats (Loiseau et al., 2008; Meneses et al., 2008). Controversially, it has been demonstrated that 5‐HT6R agonist may also enhance cognition, but the mechanism behind this paradoxical finding is poorly understood (Kendall et al., 2011; Ramirez, 2013). Due to early positive findings, several 5‐HT6R antagonists are currently being tested in clinical trials in Alzheimer's disease (Wicke et al., 2015). Larger phase‐II/III trials have failed to demonstrate any statistically significant impact on cognition (Hung & Fu, 2017; Khoury et al., 2018). However, despite mounting evidence for the therapeutic value of 5‐HT6R, the role of 5‐HT6R in learning and memory ability is very complex and not fully understood.

5‐HT6R is considered to be specifically concentrated in the membrane of the primary cilia in neurons (Brailov et al., 2000). The primary cilia are membrane‐bound, centriole‐derived projections from the cell surface that contain a microtubule cytoskeleton, known as the ciliary axoneme, surrounded by a ciliary membrane and basal body (Yuan et al., 2015). The primary cilia detect physical and chemical cues in the environment, including light (in photoreceptor cells), mechanical forces, growth factors, and neurotransmitters (Youn & Han, 2018). They also play critical roles in Sonic Hedgehog (SHh) signaling (Gazea et al., 2016), which is vital to development, such as axis formation, neuronal differentiation, bone morphogenesis (Bastida et al., 2009), and parathyroid hormone and retinoid syntheses (Bertrand & Dahmane, 2006; Deschaseaux et al., 2009). To date, various studies have demonstrated that ciliary G protein‐coupled receptors (GPCRs), including 5‐HT6R, affect the primary cilia morphology and various neuronal functions (Einstein et al., 2010; Higginbotham et al., 2012; Mukhopadhyay et al., 2013; Tomoshige et al., 2017). However, the mechanism by which 5‐HT6R affects primary cilia morphology and function is still unclear and the role of the primary cilia in the central nervous system (CNS) is not fully understood.

In the brain, 5‐HT6R is widely expressed and highly enriched in the caudate‐putamen, nucleus accumbens, and olfactory tubercle, with lower expression in the hippocampus, cortex, and amygdala (Bonasera et al., 2006; Heal et al., 2008). However, the function of 5‐HT6R in the CNS has not been fully elucidated. The Bonasera group deleted a 300 bp segment encoding a portion of 5‐HT6R protein in 2006 (Bonasera et al., 2006). However, the animals exhibited no obvious abnormalities in their baseline behaviors (Bonasera et al., 2006), possibly because the remaining fragment retained part of the 5‐HT6R function. Here, we utilized a gene‐targeting approach to generate mice constitutively lacking 5‐HT6R. Although loss of 5‐HT6R appeared to have no effect on general motor ability, remarkable abnormalities were observed in learning and memory ability as well as in anxiety level. 5‐HT6R knockout (5‐HT6R^−/−^) mice showed significant ciliary defects, including decreased cyclic adenosine monophosphate (cAMP) levels and abnormalities in the Sonic Hedgehog (SHh) signaling pathway. We found that 5‐HT6R deficiency was also related to changes in neuronal morphology, including dendrite complexity and axon initial segment (AIS) location. Neurons lacking 5‐HT6R exhibited increased neuronal excitability.

## 2 RESULTS

### 2.1 Anxiety and cognitive impairments were observed in 5‐HT6R^−/−^ mice

5‐HT6R gene was disrupted by inserting a creERT2‐P2A‐EGFP‐SV40PA expression cassette at the exon 2 (Figure S1A). Homologous recombination of the targeting vector and germline insertion of the disrupted locus were confirmed by Southern blotting (Figure S1B). Western blot analysis of 5‐HT6R confirmed that the null mutant mice were devoid of 5‐HT6R (Figure S1C). Heterozygote crosses resulted in wild‐type (WT), heterozygous, and homozygous mutant mice, consistent with Mendelian ratios and indicating that the mutation did not impair embryonic or postnatal viability. 5‐HT6R^−/−^ mice were fertile. There was no visible difference between the 5‐HT6R^−/−^ mice and their WT siblings in appearance and body weight (Figure S1D). Loss of 5‐HT6R also appeared to have no effect on general motor ability, as indicated by the rotarod test results (Figure S2A‐B).

**FIGURE 1:**
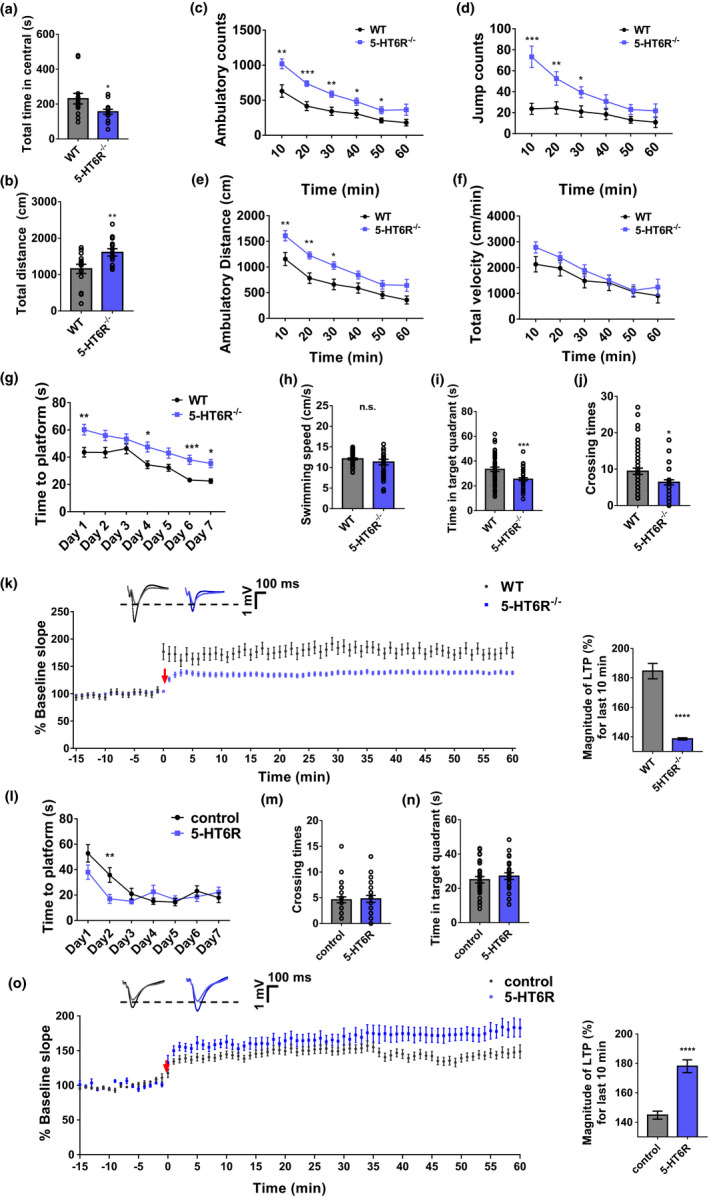
Anxiety and cognitive impairment were induced in 5‐HT6R^−/−^ mice. (a–b) 5‐HT6R^−/−^ mice spent (a) less time in the central area (*p* = 0.0335) and (b) traveled greater distances (*p* = 0.0091) in the open field. (c–f) 5‐HT6R^−/−^ mice had fewer (c) ambulatory counts (two‐way ANOVA, interaction; *F*_5, 130_ = 2.483, *p* = 0.0349; time *F*_5, 130_ = 47.36, *p* < 0.0001; gene, *F*_1, 26_ = 14.99, *p* = 0.0007), (d) fewer jump counts (two‐way ANOVA, interaction; *F*_5, 130_ = 6.415, *p* < 0.0001; time *F*_5, 130_ = 16.92, *p* < 0.0001; gene, *F*_1, 26_ = 11.05, *p* = 0.0026), and (e) shorter ambulatory distances (two‐way ANOVA, interaction; *F*_5, 130_ = 1.385, *p* = 0.2341; time *F*_5, 130_ = 54.26, *p* < 0.0001; gene, *F*_1, 26_ = 10.3, *p* = 0.0035) in the open field, (f) but there was no significant difference in average velocity between the two groups during the entire experiment (two‐way ANOVA, interaction, *F*_5, 130_ = 0.9234, *p* = 0.4681; time *F*_5, 130_ = 24.05, *p* < 0.0001; gene, *F*_1, 26_ = 1.248, *p* = 0.2742 n = 14 for each group). (g) In the Morris water maze test, the time to the platform was significantly longer for 5‐HT6R^−/−^ mice than for WT mice (Day 1, *p* = 0.0072, Day 4, *p* = 0.0301, Day 6, *p* = 0.0001, Day 7, *p* = 0.0377; WT: n = 18, 5‐HT6R^−/−^: n = 14). (h–j) Though there was no significant difference between 5‐HT6R^−/−^ mice and WT mice in (h) swimming speed (*p* = 0.2183; WT: n = 18, 5‐HT6R^−/−^: n = 14), 5‐HT6R^−/−^ mice spent less time in the target quadrant (*p* = 0.0005) and had fewer crossings (*p* = 0.0100) in the Morris water maze. (k) LTP was significantly attenuated in 5‐HT6R^−/−^ mice. Right: Summary graph shows the magnitude of LTP measured during the last 10 min post‐induction (51–60 min) in WT and 5‐HT6R^−/−^ hippocampal slices (WT, 184.7% ± 1.7%; 5‐HT6R^−/−^, 138.6% ± 0.2%; *p* < 0.0001, *t* test). (l) In the Morris water maze test, the time to the platform decreased more quickly (Day 2, *p* = 0.0096) in mice treated with AAV‐CMV‐htr6‐EFS‐tag BFP than in the control group. (m, n) The number of crossings and time in the target quadrant showed no significant differences between the two groups. (o) LTP was partially rescued in the 5‐HT6R group. Right: Summary graph shows the magnitude of LTP measured during the last 10 min post‐induction (51–60 min) in hippocampal slices from the control and 5‐HT6R groups (Control, 144.8% ± 0.9%; 5‐HT6R, 178.0% ± 1.4%; *p* < 0.0001, *t* test)

5‐HT and its receptors have been implicated in the pathogenesis of anxiety and related mood disorders (Graeff et al., 1996; Pytliak et al., 2011). We therefore investigated the anxiety levels in 5‐HT6R^−/−^ mice using an open‐field test. 5‐HT6R^−/−^ mice spent less time in the central area (Figure 1A). The total distance traveled in the open field increased during the first 10 min (Figure 1B). The ambulatory counts (Figure 1C), jumping counts (Figure 1D), and ambulatory distance (Figure 1E) for 5‐HT6R^−/−^ mice were significantly higher than those for WT mice. The average velocity was not different between the 5‐HT6R^−/−^ and WT mice (Figure 1F), excluding the possibility that the differences observed in travel time and distance were due to the running velocity.

To investigate the possible changes in cognition in 5‐HT6R^−/−^ mice, we employed a Morris water maze and Y maze to evaluate learning and memory. The Morris water maze, a reliable test that is strongly correlated with hippocampal synaptic plasticity and N‐methyl‐D‐aspartate (NMDA) receptor function, is a classic test used to assess spatial learning and memory in rodents (Vorhees & Williams, 2006). No significant difference in swimming speed was observed between 5‐HT6R^−/−^ and WT mice (Figure 1H). 5‐HT6R^−/−^ mice displayed cognitive impairment as they took longer to find the platform (Figure 1G). 5‐HT6R^−/−^ mice also spent less time in the target quadrant (Figure 1I) with fewer crossings in the original platform location (Figure 1J). 5‐HT6R^−/−^ mice spent more time and traveled a greater distance in the IV quadrant (Figure S2C‐D). In recent years, more automated Y‐maze applications have been used to evaluate continuous spontaneous alternation in rats and mice (Heredia‐López et al., 2016; Morellini, 2013). No significant difference was observed in the arm entries between WT and 5‐HT6R^−/−^ mice in the Y‐maze test (Figure S2E). Long‐term potentiation (LTP) is associated with synaptic alterations and learning/memory (C. S. Huang et al., 2005). To investigate whether the cognitive deficits in 5‐HT6R^−/−^ mice were associated with altered hippocampal synaptic plasticity, LTP was induced through high‐frequency stimulation (HFS) at the hippocampal Schaffer collateral (SC) pathway in 5‐HT6R^−/−^ mice and their WT littermate controls. Field excitatory postsynaptic potentials (​fEPSPs) evoked at the CA1 region were recorded. LTP was defined when the field EPSPs were over 20% of the baseline and lasted for at least 1 h. LTP was significantly attenuated in 5‐HT6R^−/−^ mice, evidenced by a decrease in the magnitude of LTP compared with that in WT mice (Figure 1K).

The hippocampus is a critical area for cognitive processes such as episodic memory and spatial navigation. To further confirm that 5‐HT6R depletion contributed to the cognitive impairment observed in the null mice, we delivered exogenous 5‐HT6R to the brains of null mice by injecting mCherry plasmids packaged in adeno‐associated virus (AAV) and tagged with blue fluorescent protein (BFP) (AAV‐CMV‐MCS‐EFS‐tag BFP as the vector control and AAV‐CMV‐htr6‐mCherry‐EFS for 5‐HT6R) into the hippocampus of 5‐HT6R^−/−^ mice. 5‐HT6R was successfully expressed in the 5‐HT6R^−/−^ mice hippocampus (Figure S4A). Notably, compared with the vector control group, mice with 5‐HT6R expression showed significant improvements in spatial memory. They quickly found the hidden platform on Day 2 in comparison with the control group (Figure 1L). However, there was no significant difference in the number of crossings and time in the target quadrant between the two groups (Figure 1M‐N). We also examined hippocampal LTP after the injection of AAV‐CMV‐MCS‐EFS‐tag BFP (control) or AAV‐CMV‐htr6‐EFS‐tag BFP (5‐HT6R). As expected, the level of LTP was partially rescued in the mice with 5‐HT6R delivery (Figure 1O). These results demonstrated that 5‐HT6R in the hippocampus has an important impact on learning and memory.

### 2.2 Differential gene expression was observed in 5‐HT6R^−/−^ mice

**FIGURE 2:**
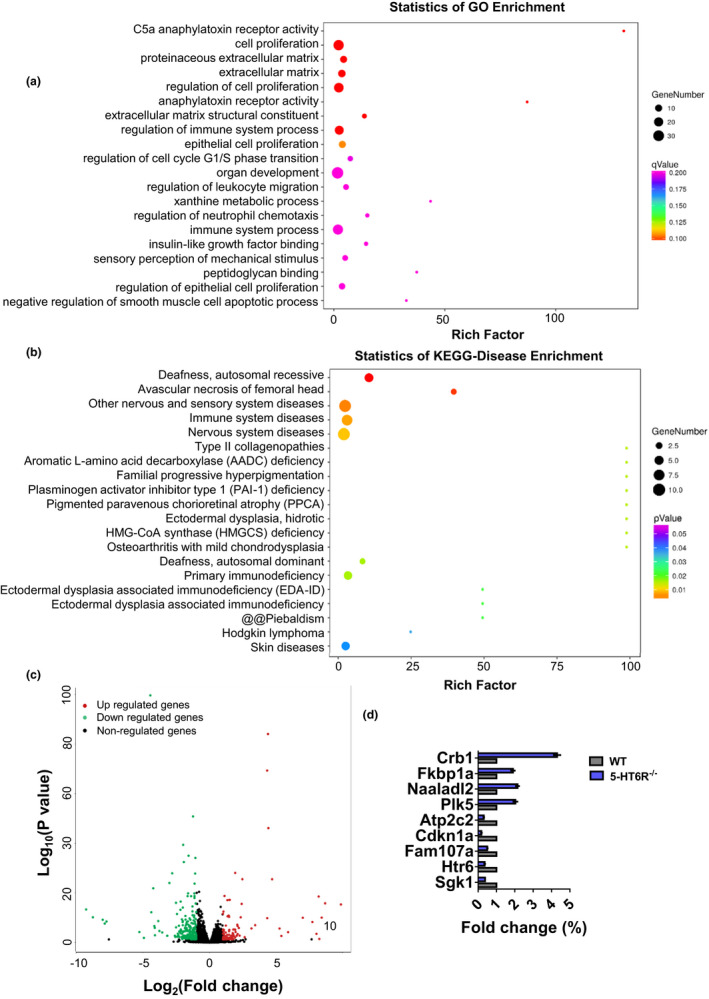
Loss of 5‐HT6R disrupts genes necessary for biological processes. (a) Gene ontology is shown for genes downregulated at P90, and the complete gene ontology results are provided in Table S3. (b) Disease ontology is shown for downregulated genes at P90, and the complete disease ontology results are provided in Table S4. (c) Volcano plot showing genes that were significantly differentially expressed (*p*‐value < 0.05) in 5‐HT6R^−/−^ mice (n = 3 for each group). (d) qRT‐PCR was used to validate the DEGs predicted by RNA sequencing

Aiming to identify potential targets resulting from the depletion of 5‐HT6R and potential mechanistic pathways involved in cognitive impairment in an unbiased manner, we performed RNA sequencing and compared the mRNA profiles of hippocampal tissues from 3‐month‐old adult WT and 5‐HT6R^−/−^ mice (Figure 2). Among 27698 genes investigated, we found 429 differentially expressed genes (DEGs; |Log_2_ Fold Change (FC)|>1, *p* < 0.05) in 5‐HT6R^−/−^ mice compared with their WT littermates (Figure 2C; Table S1). Some of the genes of interest, such as *Tekt4*, *Tppp3*, *Sgk1*, *Cdkn1a*, and *Fam107a*, were among the DEGs. The gene product of *Tekt4* is an important component of the flagella (cilia) in sperms (Roy et al., 2007). *Tppp3* can positively adjust microtubule assembly (Zhou et al., 2010). *Sgk1* expression products are related to NMDA receptors (Xiao et al., 2019) and affect neuronal proliferation, migration, and excitability (Shin et al., 2012). *Fam107a* is involved in neurite growth, neurodevelopment, and synaptic plasticity (Wang et al., 2019). The *Fam107a* gene can alter actin dynamics and cognition (Kretzschmar et al., 2018). Our results suggested that various genes involved in cilia formation, synaptic plasticity, and neuronal excitability were altered in 5‐HT6R^−/−^ mouse tissues.

In order to categorize the molecular pathways for these DEGs, gene ontology (GO) analysis was performed using the DAVID online toolbox. The top 20 enriched GO terms for the DEGs (Figure 2A), according to the percentage of genes, indicated extensive associations with cell proliferation, the proteinaceous extracellular matrix, the extracellular matrix, and extracellular matrix structural constituents. It has been reported that 5‐HT6R is predominantly located in the primary cilia and profoundly affects the morphology of the cilia (Brailov et al., 2000; Guadiana et al., 2013; Hu et al., 2017). Primary cilia contain various receptors for extracellular ligands (Guemez‐Gamboa et al., 2014). Previous studies have shown that ciliary GPCRs play a crucial role in the maintenance and regulation of the morphology (Guadiana et al., 2013) and receptor localization of the cilia (Guadiana et al., 2013), as well as their downstream cellular effectors (Mukhopadhyay et al., 2013). Overexpression of 5‐HT6R in neurons induced elongated and often branched primary cilia (Guadiana et al., 2013). Therefore, we suspect that deletion of 5‐HT6R may affect functions of the primary cilia.

We next investigated whether 5‐HT6R loss altered genes implicated in any human diseases. Disease ontology (DO) analysis was performed using the DAVID online toolbox. Analysis of DO terms revealed DEGs associated with nervous system diseases as well as other nervous and sensory system diseases (Figure 2B). Recent studies have indicated that 5‐HT6R may be a potential target for cognitive improvement in AD (Hu et al., 2017; Khoury et al., 2018; Ramirez, 2013), schizophrenia (de Bruin & Kruse, 2015), and other cognitive or memory deficits. Abnormal cilia length has been observed in several neurological disorders (Chakravarthy et al., 2012; Formichi et al., 2018; Hu et al., 2017). These results suggest the potential roles of 5‐HT6R in a variety of neurological diseases and ciliopathies.

### 2.3 5‐HT6R mutant mice showed ciliary defects and decreased cAMP levels

**FIGURE 3:**
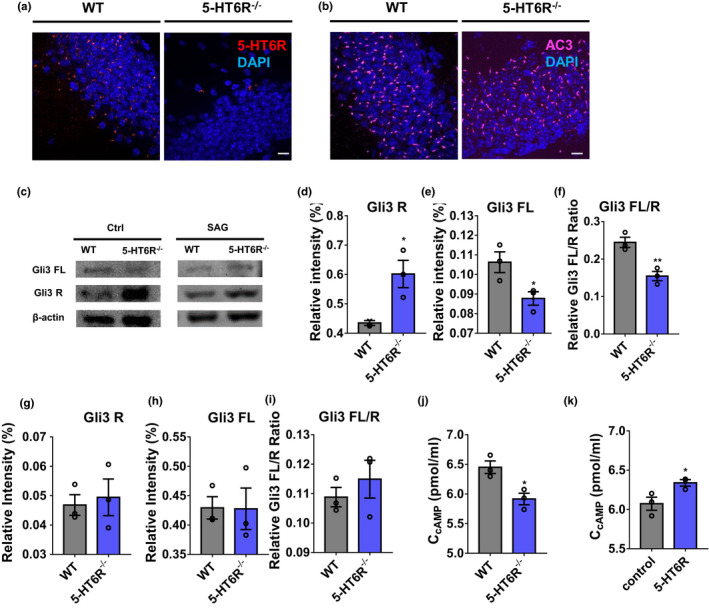
5‐HT6R mutant mice showed ciliary defects and decreased cAMP levels. (a) Immunostaining of 5‐HT6R was performed in the hippocampus DG of WT and 5‐HT6R^−/−^ mice. Scale bar: 10 μm. (b) The appearance of the cilia was normal in the DG of WT and 5‐HT6R^−/−^ mice. Immunostaining with anti‐AC3 antibody revealed no gross disruption in the ciliary structure or number in 5‐HT6R^−/−^ mice. Scale bar: 10 μm. (c) Gli3FL and Gli3R expression in the hippocampus of WT or 5‐HT6R^−/−^ mice treated with (right) or without (left) SAG. (d‐f) 5‐HT6R^−/−^ mice expressed more Gli3R (*p* = 0.0032) and less Gli3FL (*p* = 0.0228) in the hippocampal lysate compared with WT mice. (g–i) After treatment with SAG, the expression of Gli3FL (*p* = 0.7336) and Gli3R (*p* = 0.9695) remained at the same levels in WT and 5‐HT6R^−/−^ mice (n = 3 from three mice in each group). (j) The cAMP level in the hippocampus of 5‐HT6R^−/−^ mice was significantly lower than that in WT mice (*p* = 0.0208; n = 3 from three mice in each group). (k) The cAMP level in the hippocampus of the control group was significantly lower than that in the 5‐HT6R group (*p* = 0.0460; n = 3 from three mice in each group)

Based on our RNA‐seq results, we aimed to determine whether depletion of 5‐HT6R altered ciliary signaling and function. Using the 5‐HT6R antibody, not surprisingly, we did not detect 5‐HT6R‐positive primary cilia in 5‐HT6R^−/−^ mice (Figure 3A). Type 3 adenylyl cyclase (AC3), an established marker for the cilia, was applied to detect the presence and morphology of primary cilia (Figure 3B). We found no difference in the length of the primary cilia in the hippocampus of WT and 5‐HT6R^−/−^ mice (Figure S1E–F). The primary cilia also play critical roles in SHh signaling (Gazea et al., 2016), which is vital to development, such as axis formation, neuronal differentiation, bone morphogenesis (Bastida et al., 2009), parathyroid hormone and retinoid syntheses (Bertrand & Dahmane, 2006; Deschaseaux et al., 2009). Gli3, a member of the Gli family and a key intracellular transcription factor in the SHh signaling pathway (Bai et al., 2004), was measured in the hippocampus of WT and 5‐HT6R^−/−^ mice. In the absence of SHh signaling, most full‐length Gli3 (Gli3FL) are hydrolyzed by protein kinase A into a 75 kDa transcriptional repressor (Gli3R). We found that the Gli3FL was significantly decreased while Gli3R was dramatically elevated in 5‐HT6R^−/−^ mice (Figure 3C–F). Selleck's Smoothened (Smo) Agonist (SAG) is a small‐molecule agonist that promotes Smo protein from the cell membrane to the ciliary membrane, thereby activating transcription factors downstream of the SHh signaling pathway (Chen et al., 2002). SAG is cell‐permeable and can cross the blood–brain barrier to activate the SHh signaling pathway in mice. After intraperitoneal SAG injection, the expression of Gli3FL and Gli3R remained similar in WT and 5‐HT6R^−/−^ mice (Figure 3C, G–I). These results suggested that ciliary signaling was affected in 5‐HT6R^−/−^ mice neurons. 5‐HT6R is a G_s_‐coupled receptor that activates cAMP (Dayer et al., 2015). We found that the cAMP levels in 5‐HT6R knockout mouse neurons were significantly lower than those in WT mice (Figure 3J). We also examined hippocampal cAMP levels after the injection of AAV‐CMV‐MCS‐EFS‐tag BFP (control) or AAV‐CMV‐htr6‐EFS‐tag BFP (5‐HT6R). As expected, the level of cAMP was partially rescued in mice with 5‐HT6R expression (Figure 3K).

### 2.4 5‐HT6R^−/−^ mice showed dendritic and synaptic alterations

**FIGURE 4:**
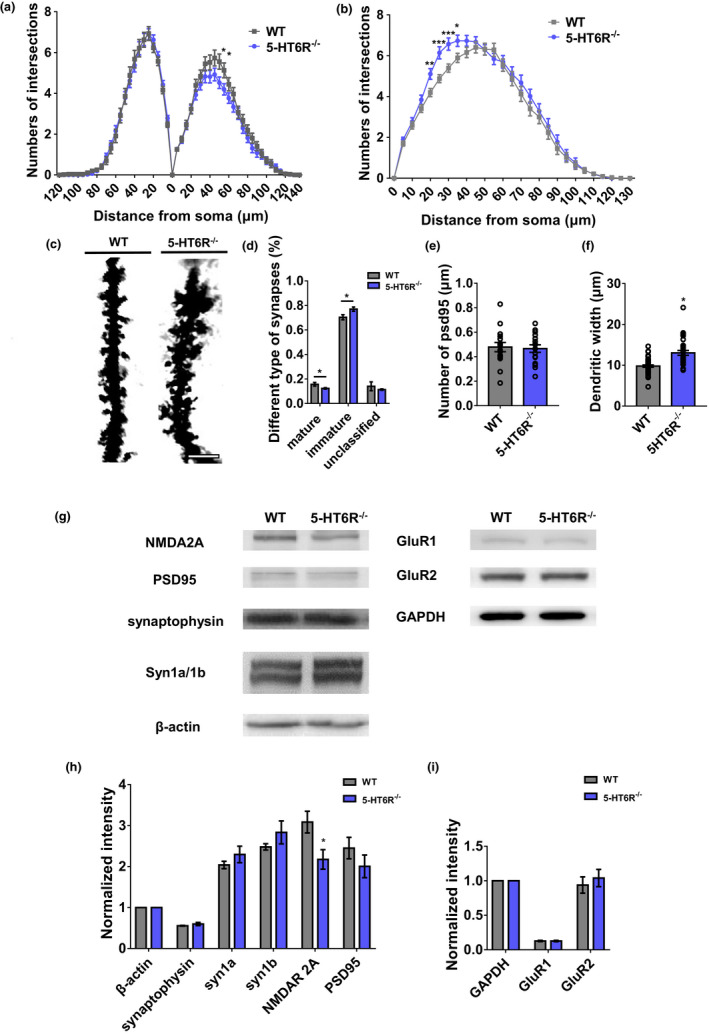
5‐HT6R^−/−^ mice showed dendritic and synaptic alterations. (a, b) The dendritic complexity of pyramidal neurons in (a) CA1 (apical 50 μm, *p* = 0.0269; 55 μm, *p* = 0.0423) and (b) the DG hippocampus (20 μm, *p* = 0.0027; 25 μm, *p* = 0.00016; 30 μm, *p* = 0.00054; 35 μm, *p* = 0.01057) is shown. (c) The dendrite of neurons following Golgi staining in the hippocampus of WT and 5‐HT6R^−/−^ mice. Scale bar: 5 μm. (d) The percentage of each spine shape in the hippocampus neurons (mature synapses, *p* = 0.02411; immature synapses, *p* = 0.013012; unclassified synapses, *p* = 0.274577; WT: n = 47, 5‐HT6R^−/−^: n = 33). (e) There was no significant difference between WT and 5‐HT6R^−/−^ mice in the number of PSD95 (*p* = 0.8029; n = 16 for each group). (f) The dendritic width was greater in 5‐HT6R^−/−^ mice (*p* < 0.0001, WT: n = 47, 5‐HT6R^−/−^: n = 33). (g–i) NMDAR2A (*p* = 0.0284), but not PSD95, synapsin1a/b, GluR1/2, or synaptophysin, was significantly downregulated in the hippocampus of 5‐HT6R^−/−^ mice (n = 6 from three mice in each group)

Besides regulatory genes in cilia morphology and signaling, many of the DEGs were related to synaptic function and plasticity. To investigate alterations in the synapses, the dendrites of pyramidal neurons in the dentate gyrus (DG) and CA1 areas of the hippocampus were quantified with golgi‐stained brain sections from 5‐HT6R^−/−^ and WT mice. Dendrite morphogenesis is a complex but well‐regulated process that includes the development of dendritic branches, forming the characteristic dendrite arbors and dendritic spines and allowing neurons to communicate with each other (Kulkarni & Firestein, 2012). Dendritic complexity is a critical determinant of neuronal connectivity (Karlsgodt et al., 2008). Sholl analysis was performed to obtain a broader picture of the dendritic complexity. Overall, we found that 5‐HT6R null mutations reduced the complexity of the CA1 pyramidal neurons (Figure 4A, S5A) and, in contrast, increased the complexity of the DG granule cells (Figure 4B, S5A). The synapse is the fundamental functional unit of the brain. Neurons communicate with each other through synapses. The morphology of single dendritic spines directly affects the local function of synapses (Fifková, 1985). Large, mushroom‐shaped dendritic spines are associated with mature, stabilized synapses and memory formation, whereas thin or filopodia‐like spines are associated with weaker or developing, less‐mature synapses (Tan & Waxman, 2015). We classified dendritic spines as “mature” and “immature” based on these characteristics. There was a remarkable decrease in the proportion of mature synapses and an increase in the proportion of immature synapses (Figure 4C–D). Postsynaptic density protein 95 (PSD95) is the most abundant scaffold protein in mature synapses (Jeyifous et al., 2016). There was no significant change in the number of mature synapses labeled by PSD95 in cultured hippocampal neurons (Figure 4E). This difference in the ratio of mature to immature synapses is most likely due to the growth of immature synapses. Moreover, the dendritic width in 5‐HT6R^−/−^ mice was significantly broader (Figure 4F), possibly due to the presence of many immature dendritic spines.

As postsynaptic receptors are critical for mature synaptic function, it is important to determine whether 5‐HT6R has any effect on the expression levels of proteins that are involved in synaptic plasticity. LTP has been associated with synaptic plasticity for a long time. Postsynaptic mechanisms are known to be involved in LTP (Liu et al., 2019), and NMDA receptors play an important role in synaptic plasticity in the adult brain (Sachser et al., 2017). NR2A or NR2B combined with NR1 are the subunits of NMDA receptors. Accordingly, we evaluated the protein expression of the NR2A NMDA subunit in the hippocampus of WT and 5‐HT6R^−/−^ mice. We found that the NR2A expression level was significantly downregulated in 5‐HT6R^−/−^ mice hippocampus (Figure 4G–H). PSD95 is involved in synaptic plasticity and dendritic spine morphogenesis and mediates NMDA receptor clustering and function at postsynaptic terminals (Coley & Gao, 2018). No statistical difference was found in the PSD95 expression levels between the hippocampus of WT and 5‐HT6R^−/−^ mice (Figure 4G–H). We also evaluated the expression of the α‐amino‐3‐hydroxy‐5‐methyl‐4‐isoxazolepropionic acid (AMPA) receptors GluR1 and GluR2, which showed no significant differences between WT and 5‐HT6R^−/−^ mice (Figure 4G, I). These results suggested that 5‐HT6R may affect neuronal function by regulating the expression of synaptic proteins in the hippocampus.

### 2.5 Neuronal excitability and AIS changed in 5‐HT6R^−/−^ mice

**FIGURE 5:**
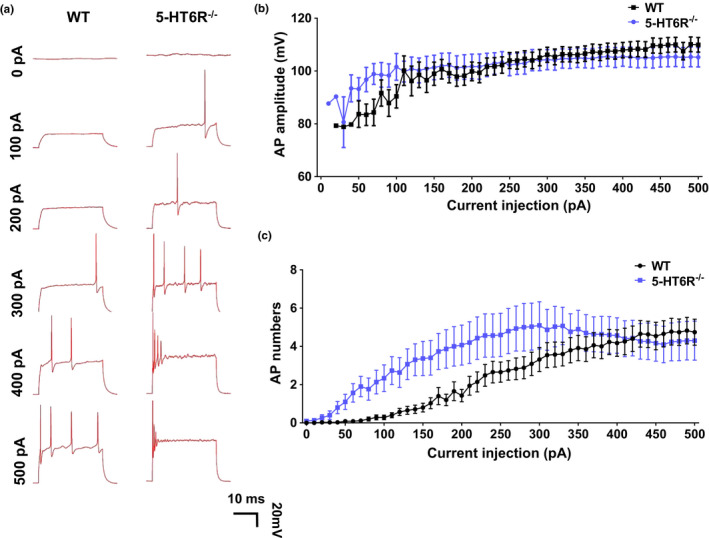
Neuronal excitability changed in 5‐HT6R^−/−^ mice. (a) WT and 5‐HT6R^−/−^ mice showed different voltage responses to different current pulses (0–500 pA) in hippocampus pyramidal neurons. (b, c) 5‐HT6R^−/−^ mice showed a significant increase in (c) AP numbers (two‐way ANOVA, interaction: *F*_50, 3213_ = 1.094, *p* = 0.3024; current injection, *F*_50, 3012_ = 9.217, *p* < 0.0001; gene, *F*_1, 3213_ = 73.25, *p* < 0.0001), but not (b) AP amplitudes compared to WT mice (Multiple T test, All groups: *p* > 0.05, WT: n = 35, 5‐HT6R^−/−^: n = 29)

Neuronal excitability is a critical determinant of dendritic complexity during the assembly of sensory pathways (Frangeul et al., 2017). In some cells, dendrite growth and remodeling continue into adulthood (Prigge & Kay, 2018). Neuronal activity is important for the elaboration and stabilization of dendrite branches (Prigge & Kay, 2018). Therefore, it is necessary to determine the excitatory changes in individual hippocampal neurons. To study the excitatory changes, we performed a whole‐cell patch‐clamp experiment with hippocampal neurons. The excitability of neurons varies in distinct cell types. Based on the cell membrane capacity, the neurons we examined mostly appeared to be the same types of cells (Figure S3A). We injected 500‐ms‐duration ramp currents with amplitudes ranging from 10 to 500  pA. Surprisingly, the patch‐clamp recording showed no difference in the action potential (AP) amplitudes in the mutants compared to those in the WT mice (Figure 5). However, the neuronal excitability in 5‐HT6R^−/−^ mice had changed to fire more APs with greater frequency (Figure 5A–C).

**FIGURE 6:**
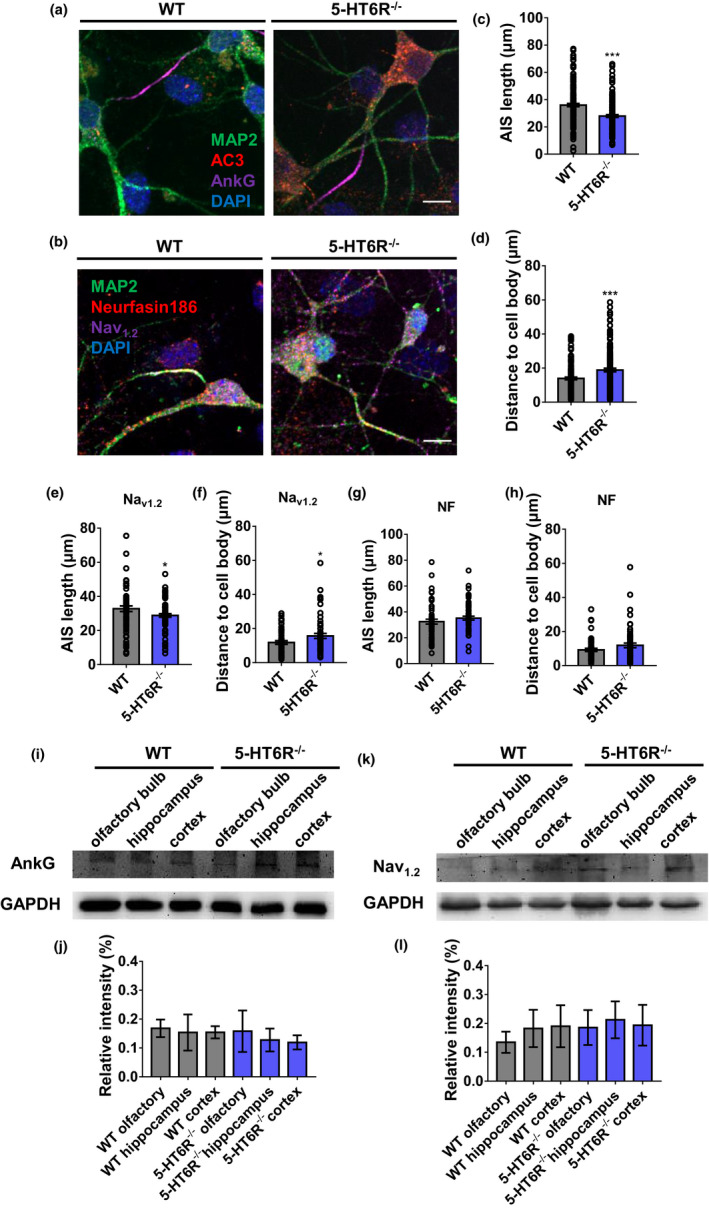
Axon initial segment structure changed in 5‐HT6R^−/−^ mice. (a, b) Immunocytochemistry results for MAP2, AC3, AnkG, NF186, and Na_v__1.2_ are shown in the neurons of WT and 5‐HT6R^−/−^ mice. Scale bar: 10 μm. (c, d) The AIS was marked by AnkG staining. 5‐HT6R^−/−^ mice possessed shorter AIS than WT mice (*p* < 0.001). 5‐HT6R^−/−^ mice had a longer distance between the AIS and soma than WT mice (*p* = 0.0055; WT: n = 71, 5‐HT6R^−/−^: n = 61). (e, f) 5‐HT6R^−/−^ mice AIS based on Na_v__1.2_ moved away from the soma (*p* = 0.0148) and became shorter (*p* = 0.0124) than that of WT mice (WT: n = 81, 5‐ HT6R^−/−^: n = 83). (g, h) 5‐HT6R^−/−^ mice AIS based on NF186 showed no differences between 5‐HT6R^−/−^ mice and WT mice (AIS length, *p* = 0.6839; AIS distance, *p* = 0.1623; WT: n = 57, 5‐HT6R^−/−^: n = 60). (i–l) There was no significant difference in the expression level of (i, j) AnkG and (k, l) Na_v__1.2_ in the olfactory bulb, hippocampus, and cortex of WT and 5‐HT6R^−/−^ mice (n = 3 from three mice in each group)

Neurons receive synaptic inputs that converge on their dendrites and cell bodies. The summation of synaptic inputs gives rise to APs at the AIS. In neurons, the AIS is a specialized region near the start of the axon and is the site of AP initiation (Huang & Rasband, 2018). AIS plasticity is known to be crucial for the homeostatic control of neuronal excitability (Chand et al., 2015; Grubb & Burrone, 2010; Kuba et al., 2010). This excitatory change is most likely due to structural plasticity in the AIS. We measured AIS length and the distance between the AIS and cell body to examine AIS morphology in primary cultured hippocampal neurons (Figure 6A,B). AIS length was measured from the 1/3 brightest point near the soma to the 1/3 brightest point distal to the soma (Grubb & Burrone, 2010). AnkG is a crucial AIS scaffolding protein with population‐specific differences in AIS position and length (Leterrier & Dargent, 2014). 5‐HT6R^−/−^ mice possessed shorter AIS (Figure 6C) and had a longer distance between the AIS and soma than WT mice (Figure 6D). The AIS has a high density of voltage‐gated ion channels, membrane proteins, and a unique repertoire of sub‐membranous cytoskeletal scaffolds. We found that the AIS in 5‐HT6R^−/−^ mice revealed based on Na_v1.2_ moved away from the soma and became shorter than that in WT mice (Figure 6E–F). However, the AIS revealed based on NF186 showed no differences between 5‐HT6R^−/−^ and WT mice (Figure 6G–H). AIS plasticity may be mainly caused by changes in AnkG and its related proteins, and NF186 as a marker can help the AIS relocate back to its original position. Are these changes in AIS morphology caused by lower expression levels or a different distribution of these proteins? To explore the underlying mechanisms, we examined the expression levels of these proteins in the hippocampus of 5‐HT6R^−/−^ and WT mice. AnkG and Na_v1.2_ expression levels showed no differences between 5‐HT6R^−/−^ and WT mice (Figure 6I–L).

## 3 DISCUSSION

In this study, we bred 5‐HT6R knockout mice and investigated the function of 5‐HT6R in the CNS. The Bonasera group previously reported that deleting 300 bp of 5‐HT6R did not affect the baseline behaviors of the mice (Bonasera et al., 2006). Additionally, 5‐HT6R triple mutant (F69L/T70I/D72A) mice showed reduced sensitivity to diet‐induced obesity (Frassetto et al., 2008). None of these results can explain the observed effects of 5‐HT6R on cognitive function. Hence, we inserted an expression cassette at exon 2 to prevent 5‐HT6R expression. Homozygous mutant mice were born at the expected ratio, developed normally, and were fertile with no obvious differences in appearance and body weight, suggesting that 5‐HT6R is not required for normal development and growth, which is consistent with previous studies (Bonasera et al., 2006; Frassetto et al., 2008). However, the 5‐HT6R^−/−^ mice exhibited abnormal anxiety levels. Similar anxiety‐like phenomena have been observed in an elevated plus maze and a social interaction test in rats given 5‐HT6R antisense oligonucleotides (Khoury et al., 2018; Meneses et al., 2008). Other studies have shown that 5‐HT6 antagonists can improve learning and memory ability (de Bruin & Kruse, 2015; Hu et al., 2017; Routledge et al., 2000). Unlike the results observed with the antagonist, 5‐HT6R^−/−^ mice exhibited decreased learning and memory in the Morris water maze. There are several possible explanations for the different effects on cognitive behavior observed after 5‐HT6R deletion and the administration of 5‐HT6R antagonists. First, changes in behavior resulting from chronic 5‐HT6R deletion may differ from those induced by acute pharmacological treatment. Second, constitutive deletion of 5‐HT6R may trigger developmental compensations. It seems that 5‐HT6R plays opposite and complex roles at different developmental stages, and further research is needed.

We also found that although the loss of 5‐HT6R did not affect the length of the primary cilia, it did affect the SHh signaling pathway in the primary cilia. We found that Gli3FL was significantly decreased and Gli3R was dramatically increased in 5‐HT6R^−/−^ mice. The total volume of primary cilia is tiny compared to that of the cell body, but the former contains a high density of various receptors. Previous studies demonstrated that the overexpression of ciliary GPCRs, especially 5‐HT6R, induced elongation and often resulted in forked primary cilia (Guadiana et al., 2013; Hu et al., 2017). To date, various studies have shown that ciliary GPCRs are associated with primary cilia morphology and neuronal functions (Einstein et al., 2010; Higginbotham et al., 2012; Tomoshige et al., 2017). The dysfunctional primary cilia observed in the 5‐HT6R mutant mouse neuron may be attributed to an increase in Gli3Rs. This defective processing of Gli3 with aberrant SHh signaling is similar to that seen in the brains of individuals with Joubert syndrome/Meckel syndrome (Aguilar et al., 2012). Primary cilia deficiency can lead to an abnormal nervous system, including cognitive impairment (Fry et al., 2014). Children with primary ciliary dyskinesia, who have structurally and functionally abnormal cilia, show significant behavioral and social problems (Carotenuto et al., 2013). Cognitive function and memory performance in patients with Bardet–Biedl syndrome (BBS) is also a ciliopathy due to hippocampal hypoplasia (Bennouna‐Greene et al., 2011). Ciliary deficiency as well as cognition and mood disorders can be seen in ciliopathies, consistent with the findings in the 5‐HT6R^−/−^ mice. Our data suggested that 5‐HT6R affects the function of the nervous system through the primary cilia and provides inspiration for researchers to further study 5‐HT6R or the functions of the primary cilia.

5‐HT6R^−/−^ mice showed alterations in their synapses and AIS. We found that the LTP level of neurons in the hippocampus CA1 area was reduced in 5‐HT6R‐null brain slices. Further, it is believed that the 5‐HT6R agonist WAY‐181187 can modulate synaptic plasticity and long‐term enhancement in hippocampal CA1 (West et al., 2009). This effect is blocked by the 5‐HT6R antagonist SB‐399885 (West et al., 2009). 5‐HT6R regulates cortical neuronal migration and morphology (Hu et al., 2017; Lesiak et al., 2018). A previous study suggested that the modification of synaptic strength produced by LTP underlies memory storage (Lisman et al., 2018). We hypothesized that the dendrites and axons, two main neuronal structures that receive and conduct signals, may be altered by 5‐HT6R mutation. We observed an abnormal aggregation of spines, which might lead to increased dendritic width. In RNAseq, the *Fam107a* gene was found to be downregulated, which can alter actin dynamics and impact cognition (Kretzschmar et al., 2018). This finding is consistent with our results above. Additionally, variable neuropsychiatric phenotypes have been observed with abnormal dendritic spines (Martinez‐Cerdeno, 2017). We found that the NR2A expression level was significantly downregulated in 5‐HT6R^−/−^ mice hippocampus. Researchers have shown that dysfunctional NMDA receptors resulted in the down‐regulation of 5‐HT6R expression in the primary cilia in the hippocampus CA1 region, which may be related to schizophrenia (Shiwaku et al., 2017). We hypothesized that changes in the dendrites and their spines are the mechanisms underlying the cognitive impairment observed in 5‐HT6R mice. However, the neurons from 5‐HT6R^−/−^ mice were more excitable than those of WT in the whole‐cell patch, suggesting that the axons may be involved in the excitability and regulation of inhibitory homeostasis. In neurons, the AIS is a specialized region near the beginning of the axon where APs are first initiated and the AIS location is associated with changes in the electrical activity of neurons (Grubb & Burrone, 2010). Our data showed that the change in the AP firing frequency in 5‐HT6R^−/−^ mice was accompanied by the alteration of AIS protein localization, suggesting that the AIS may play an important role in regulating homeostasis after 5‐HT6R impairment. However, how the deletion of 5‐HT6R affects AIS specialized protein localization remains to be determined. Identifying the effects of changes in neuronal morphology thus appears to be critical for understanding 5‐HT6R‐related pathologic mechanisms.

In summary, our findings highlight the complexity of 5‐HT6R functions in ciliary and neuronal physiology and support the hypothesis that this receptor modulates neuronal morphology. It is increasingly clear that 5‐HT6R is a promising target for the treatment of a wide range of human diseases. We suggest that future studies and experiments should be conducted taking into consideration receptor ciliary localization and neuronal alterations to clarify the mechanisms underlying the role of 5‐HT6R. The present work may help address the mechanisms for the various physiological functions involving 5‐HT6R to explain cognitive deficits in a variety of human diseases such as AD, schizophrenia, and ciliopathies.

## 4 MATERIALS AND METHODS

### 4.1 Animals

WT C57BL/6 mice were obtained from the Peking University Animal Care and Use Committee. All animal studies were conducted in accordance with the Guide for the Care and Use of Laboratory Animals (8th edition) and approved by the Institutional Animal Care and Use Committee of Peking University. The laboratory approval number from the Association for Assessment and Accreditation of Laboratory Animal Care (AAALAC)‐approved Animal Facility at Peking University Laboratory Animal Center (LAC‐PKU) IACUC is LSC‐ZhangY‐1. The development of the *Htr6* gene‐modified mouse model (Htr6‐creERT2‐P2A‐EGFP‐SV40PA) was carried out in strict accordance with the guidelines of the Institutional Animal Care and Use Committee (IACUC) at the Shanghai Model Organism Center, Inc. The number of this study is 2015–0003–903. DNA was extracted from the tails of P0 mice using a TIANamp Genomic DNA Kit (Tiangen). The details for all primers used are provided in Table S2 as well as the details for all antibodies and instruments.

### 4.2 Measurement of cAMP levels

We used an enzyme immunoassay kit (Cayman Scientific, 501040) to measure the cAMP levels in WT, 5‐HT6R^−/−^ (control), and 5‐HT6R group mice. The hippocampus was dissected from the brain as previously described (Einstein et al., 2010).

### 4.3 Rotarod test

Mice were placed on a rotating rod (MED, ENV‐575A, USA) spinning at 4 RPM with a lane width of 50 mm and a diameter of 30 mm. Once stabilized, the mice were subjected to an incrementally increase in speed by 1 RPM per 8 s. Each animal underwent three trials. The length of time that the mice managed to remain on the rod and the speed at which they fell off the apparatus were recorded. Average values from the three trials were used for further analysis.

### 4.4 Y‐Maze test

WT and 5‐HT6R^−/−^ mice were tested in a Y maze. The apparatus consisted of three arms that were 34 cm long, 8 cm wide, and 14.5 cm high. Different images were posted at the ends of the three inner arms, consisting of a red circle, black square, and white triangle. The test began when a mouse was placed on the apparatus facing the wall. After 10 min, the experiment was terminated. The automatic tracking system (CSI) traced the position of the mouse in real time and recorded the number of times the mice visited a new arm. The arm was considered new if it differed from the previous and next arms chosen by the mouse.

### 4.5 Open‐Field test

The open‐field test was performed in another room. The apparatus used consisted of gray Plexiglas sides and a floor with the dimensions 40  ×  40  ×  30 cm. The test was initiated by placing the mice at the center of this apparatus. For the next 60 min, the movements of individual mice were recorded by a camera mounted above the apparatus. The analysis was carried out with the help of the Noldus Observer software (Ethovision 11.0). After each subsequent test, the entire apparatus was cleaned with ethanol. The center time and total distance were recorded and analyzed with Ethovision 11.0. The illumination in the testing room was set to ∼ 200 lx.

### 4.6 Morris water maze test

The water maze test consisted of a circular pool (60 cm in radius and 50 cm high) [Correction added on 4 June 2021, after first online publication: In the previous sentence, ‘60 cm in diameter’ has been corrected to ‘60 cm in radius’ in this version]. The pool was placed in water with a temperature of 21–22°C at a depth of 30 cm. The hidden platform was 11 cm in diameter and located 1.5 cm under the surface of the water. The pool was divided into four quadrants, and the hidden platform was in one of the four quadrants. Mice were tested in the four quadrants with a fixed order. The starting location in the four quadrants was random. The mice spent 90 s in the pool four times to adapt to the water environment 1 day before the experiment. The test began when a mouse was placed in one of the quadrants facing the wall and ended when the mouse got on the platform. If a mouse got on the platform within 90 s, it was allowed to remain on the platform for 10 s. If the mouse failed to get on the platform within 90 s, it was guided to the platform and allowed to stay for 10 s. The mice were trained and recorded in the four quadrants every day for 6 consecutive days to measure their swimming speed, the time to the platform, and the swimming distance to the hidden platform. On day 7, the hidden platform was removed and the time spent in the target quadrant without the platform was recorded as well as the number of crossings at the original location of the platform. The position of the mouse was traced by the automatic tracking system (CSI) in real time. All behavioral tests were conducted under red light to ensure the mice were active.

### 4.7 RNA isolation, sequencing, and data analysis

Total RNA was isolated from freshly dissected tissue using the TRNzol Reagent kit (Tiangen, DP405) according to the manufacturer's instructions. RNA was assessed with the Agilent 2100 BioAnalyzer (Agilent Technologies) and Qubit Fluorometer (Invitrogen).

Total RNA samples that met the following requirements were used in subsequent experiments: RNA integrity number (RIN) >7.0 and a 28S:18S ratio >1.8. RNA‐seq libraries were generated and sequenced by CapitalBio Technology (Beijing, China). Three biological replicates from each group were evaluated in this manner. The NEB Next Ultra RNA Library Prep Kit for Illumina (NEB) was used to construct the libraries for sequencing, and the NEB Next Poly(A) mRNA Magnetic Isolation Module (NEB) kit was used to enrich the poly(A) tailed mRNA molecules from 1 μg total RNA. The mRNA was fragmented into ~200 bp pieces. The first‐strand cDNA was synthesized from the reverse transcriptase of the mRNA fragments and random hexamer primers while the second‐strand cDNA was synthesized using DNA polymerase I and RNaseH. The end of the cDNA fragment was subjected to an end repair process that included the addition of a single “A” base, followed by ligation of the adapters. Products were purified and enriched by using polymerase chain reaction (PCR) to amplify the library DNA. The final libraries were quantified using the KAPA Library Quantification kit (KAPA Biosystems, South Africa) and an Agilent 2100 Bioanalyzer. After validation with quantitative reverse transcription‐PCR (RT‐qPCR), the libraries were subjected to paired‐end sequencing with a pair‐end 150‐bp reading length on an Illumina HiSeq sequencer (Illumina).

The human genome version of hg19 was used as a reference. The sequencing quality was assessed with FastQC (Version 0.11.5), and data with large noise were filtered using NGSQC (v0.4). The clean reads were then aligned to the reference genome using HISAT2 (Johns Hopkins University, USA) with default parameters. The processed reads from each sample were aligned against the reference genome using HISAT2 (Johns Hopkins University). Gene expression analyses were performed with Cuffquant and Cuffnorm (Cufflinks 2.2.1). Cuffdiff was used to analyze the DEGs between samples. The standardization method for Cuffdiff was geometric, with the per‐condition setting and pooled discrete model. Thousands of independent statistical hypothesis tests were conducted on the DEGs individually. The P‐value was obtained and corrected using the false discovery rate (FDR). The corrected *p*‐value (*q*‐value) was calculated using the Benjamini–Hochberg (BH) method. The *p*‐value or *q*‐value was used for significance analysis. The parameters used to classify significant DEGs were ≥twofold differences (|log_2_FC|≥1, FC: the fold change of expression) in the transcript abundance and *q* < 0.05. Annotation of the DEGs was performed based on the information obtained from the ENSEMBL, NCBI, Uniprot, GO, and KEGG databases.

### 4.8 Quantitative real‐time polymerase chain reaction

cDNA was generated by reverse transcription using the RevertAid First Strand cDNA Synthesis Kit (Thermo Scientific, K1622) according to the manufacturer's instructions. Afterward, quantitative real‐time PCR (qRT‐PCR) reactions were performed with the Tsingke Master qPCR Mix (Tsingke, TSE201) on a Roche Lightcycler 480 (Roche) according to the manufacturer's instructions. The expression of the target gene was calculated using the formula 2^−ΔΔCt^, and mRNA expression was standardized to glyceraldehyde 3‐phosphate dehydrogenase (GAPDH). All gene primer sequences are listed in Table S2.

### 4.9 Golgi staining

A modified Golgi‐Cox impregnation of neurons was performed according to the methods described in the instructions for the Hito Golgi‐Cox OptimStain™ Kit (HTKNS1125, HitoBiotec Corp). Mice were anesthetized with chloral hydrate (400 mg/kg, intraperitoneally) and perfused with 0.9% NaCl solution and 4% paraformaldehyde (PFA) in 0.1 M phosphate‐buffered saline (PBS). The mice brain was rinsed in double distilled water for 2–3 s to remove blood from the surface. Next, the mice brain was transferred to the impregnation solution, which was at least five times the volume of the brain tissue, and was stored at room temperature in the dark. The next day (after 12–24 h), the impregnation solution was replaced and the tissue was stored at room temperature (20–25°C) for 2 weeks in the dark. The brain tissue was then transferred to Solution 3, which was at least five times the volume of the brain tissue, and was stored at 4°C in the dark for 24 to 72 h. The tissue was coated in low gelling temperature agarose and was cut into 200 μm sections into double distilled water. The floating sections were mounted onto gelatin‐coated slides using a Fine Tip Natural Hair Brush. The brain slices were rinsed twice in distilled water for 3 min each time and were placed in a mixture of solutions 4 and 2 for 10 min. Next, the brain slices were rinsed twice in distilled water for 4 min each time. Then, the slices were rinsed with distilled water in 50%, 75%, and 95% ethanol for 5 min each time. Afterward, the slices were dehydrated in 100% ethanol three times for 5 min each time. Finally, the tissue was cleared in xylene twice for 5 min each time. A coverslip was applied over the sections using undiluted xylene‐based resinous mounting medium. The slide was viewed with bright field microscopy after drying.

### 4.10 Cell culture and transfection

Primary culture neurons were cultured from the hippocampus of newborn C57 mice. Fetal hippocampal samples were dissociated from the brain, cut into small pieces, and dissociated in 0.25% trypsin (Invitrogen) for 20 min. After trypsinization, an equal volume of DMEM‐F12 medium (Gibco) with 10% fetal bovine serum (FBS) (Gibco) was added and the mixture was lightly triturated until a single‐cell suspension was achieved. After 2 min of precipitation, the supernatant was collected and centrifuged at 500 × g for 2 min. The cells were resuspended in DMEM‐F12 medium with 10% FBS and plated on a coverslip coated with poly‐D‐lysine (Sigma). Neurobasal medium (Gibco) containing Pen‐Strep (Invitrogen), B27 (Gibco), and GlutaMAX (Thermo Fisher) was added to the medium after half an hour. The cells were grown in a 37°C incubator in a 5% CO_2_ environment. Half of the medium was replaced with fresh medium every 2 days.

Calcium phosphate precipitation was used for cultured cell transfection. Neurons were transfected after 3–8 days. DNA, CaCl_2_, 2× HEPES‐buffered saline (HBS), and sterile H_2_O were combined and added to each well for 1–2 h. Then, 1×HBS (pH 6.8) was added to each well to clear the precipitate. Finally, the original medium was added to each well, and the cells were returned to the 37°C incubator.

### 4.11 Western blotting

Tissue samples were collected using RIPA lysis buffer (R&D), and proteins were obtained by centrifugation. Proteins were denatured at 100°C for 5 min and separated in 10% sodium dodecyl sulfate‐polyacrylamide gel electrophoresis (SDS‐PAGE) at 80 mA for 2 h. They were then transferred to polyvinylidene difluoride (PVDF) membranes (Millipore) at 100 mA for 2 h. The membranes were blocked in 5% bovine serum albumin (BSA; Sigma) in Tris‐buffered saline (TBS; Sigma) with 0.1% Tween 20 (TBST) at room temperature for 1 h. Antibodies were diluted at a suitable concentration and added to TBST with 5% BSA. Primary antibodies were incubated overnight at 4°C. Horseradish peroxidase (HRP)‐conjugated second antibodies were added after washing three times for 10 min each time with TBST. Enhanced chemiluminescence was used to detect the optical density of HRP after washing three times for 10 min each time. BioRad ChemiDox (BioRad) was used to analyze the optical density of HRP.

### 4.12 Immunostaining

Cells were washed in PBS and fixed in 4% PFA (Sigma) for 20 min at room temperature. Then, the cells were permeabilized in 0.1% Triton at 4°C, blocked in 5% donkey serum at room temperature, and incubated with primary antibodies at 4°C for 24 h. Secondary antibodies were added to the cells for 1 h in the dark. The nuclei were stained with 4′,6‐diamidino‐2‐phenylindole (DAPI; Sigma) for 15 min. Finally, coverslips with cells were placed on slides for imaging.

### 4.13 Electrophysiological recordings

Whole‐cell patch‐clamp recordings were obtained from the soma of hippocampal neurons. The patch pipettes had a resistance of 3–6 MΩ for somatic recordings when filled with an internal solution containing (in mM) 140 potassium gluconate, 3 KCl, 2 MgCl_2_, 2 Na_2_ATP, 0.3 Na_3_GTP, 10 HEPES, and 0.2 EGTA (pH 7.2 with KOH). During recording, access resistance was monitored frequently and was compensated by up to 70%. Somatic recordings with access resistance >25 MΩ were discarded. Bridge balance and capacitance neutralization were carefully adjusted before and after every experimental protocol.

A commercial 64‐channel multisite recording system (MED64, Panasonic Alpha‐Med Sciences) was used for extracellular field potential recordings in this study. The procedures for the preparation of the MED were almost the same as described previously (Huang et al., 2006). The size of each electrode in the array was 50 × 50 μm, and all 64 electrodes were arranged in an 8 × 8 square pattern with an inter‐electrode distance of 300 μm to cover a total area of 4.4 mm^2^. Before use, the surface of the MED64 probe was treated with 0.01% poly‐L‐ornithine (Sigma, P4957) overnight at 4°C. Afterward, the probe surface was rinsed three times with sterile distilled water before immediate use in each experiment. The current sources and sinks for all 64 electrodes were transformed into two‐dimensional current source density images by bilinear interpolation at each point. After incubation, one brain slice was transferred to the prepared MED64 probe and perfused with oxygenated (95% O_2_ and 5% CO_2_) artificial cerebrospinal fluid (ACSF) at 28–30°C at a 2 ml/min flow rate. Current pulse stimulation (1–10 mA, 0.2 ms) was applied to the stimulation channel and the intensity was adjusted so that a half‐maximal field fEPSP was elicited in the channels closest to the stimulation site. The channels with fEPSPs were considered active, and their fEPSPs responses were sampled every 1 min. For every slice, we selected the maximum fEPSP from the SC pathway for analysis (Liu et al., 2011; Miao et al., 2019; Song et al., 2017). The “slope” indicated the average slope for each fEPSP recorded in the active channels. Stable baseline responses were first recorded until the baseline response variation was less than 5% within 0.5 h in most of the active channels.

### 4.14 Blue fluorescent protein‐adeno‐associated virus (BFP‐AAV) AND 5‐HT6R‐mcherry‐adeno‐associated virus construction (AVS)

Kirk Mykytyn (Ohio State University, USA) generously provided the mouse EGFP‐tagged 5‐HT6R plasmid. The sequence for a 5‐HT6R‐mCherry plasmid was constructed into a pHS‐AVC‐LW1043 vector (Syngen Tech.) to generate 5‐HT6R‐mCherry plasmids. These plasmids were packaged into pAd recombinant adeno‐associated virus (AAV) to create AAV‐CMV‐5‐HT6R‐mCherry‐EFS‐tag BFP (1 × 10^12^ vg/mL). AAV‐CMV‐MCS‐EFS‐tag BFP (1 × 10^12^ vg/mL) served as the control. We verified these constructs through sequencing.

### 4.15 Stereotactic injection

The mice were anesthetized and injected with 2 μL of AAV‐CMV‐5‐HT6R‐mCherry‐EFS‐tag BFP or AAV‐CMV‐MCS‐EFS‐tag BFP in the hippocampus for AAV vector transfection. This was done using a microinjector attached to a microinjection pump and fixed to a stereotactic instrument (RWD Life Science). The mice were placed on a warming blanket maintained at 37°C until they were fully awake. For the mice subjected to behavioral tests, the control and 5‐HT6R groups were bilaterally injected at 2 months of age, and the mice were tested behaviorally at 3 months of age.

### 4.16 Statistical evaluation

Data were collected and analyzed using GraphPad Prism 7.0 software for statistical analysis. All values represent the mean ±SEM. Statistical significance between groups was assessed with Student's t test. A *p*‐value less than 0.05 indicated statistical significance. *P*‐values were calculated with Student's *t* test. The symbols used are as follows: *p < 0.05, **p < 0.01, and ***p < 0.001.

## CONFLICT OF INTEREST

All authors declare no actual or potential conflicts of interest, including any financial, personal, or other relationships, with other people or organizations within three years of beginning the work submitted that could inappropriately influence (bias) their work.

## AUTHOR CONTRIBUTIONS

Z.S., B.W., and C.C. performed all experiments and analyzed the data. L.C. helped on animal behavioral tests. Y.Z. conceptualized the study, performed analyses, and drafted the manuscript.

### Open Research Badges

This article has earned an Open Data Badge for making publicly available the digitally‐shareable data necessary to reproduce the reported results. The data is available at https://www.ncbi.nlm.nih.gov/geo/query/acc.cgi?acc=GSE137942.

## Supporting information

Fig S1‐5

Table S1

Table S2

Table S3

Table S4

## Data Availability

The mRNA differential expression data were uploaded to NCBI Sequence Read Archive (SRA) GSE137942. https://www.ncbi.nlm.nih.gov/geo/query/acc.cgi?acc=GSE137942.
