# Correction to: 5‐HT6R null mutation induces synaptic and cognitive defects

**DOI:** 10.1111/acel.13777

**Published:** 2022-12-30

In the published version of Sun et al. (2021), the word ‘mutation’ in the article title was misspelled and the article title should read as follows.

**5‐HT6R null mutation induces synaptic and cognitive defects**

We apologize for this error.
