# Supplementary figures and images for: 5‐HT6R null mutatrion induces synaptic and cognitive defects
Source: Aging Cell. 2021 May 7;20(6):e13369. doi: 10.1111/acel.13369 (PMC8208783; doi:10.1111/acel.13369)

Fig.S1

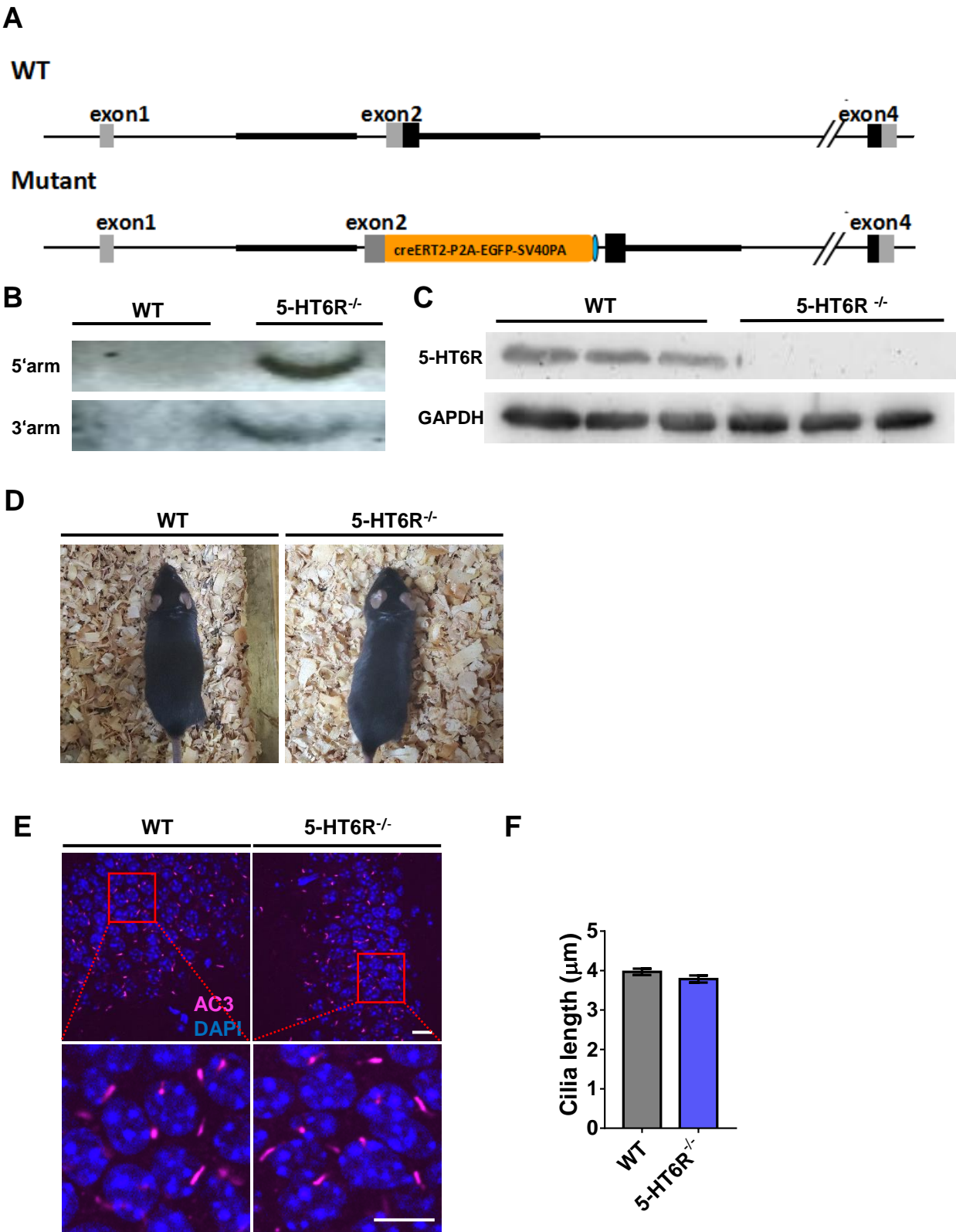

Fig.S2

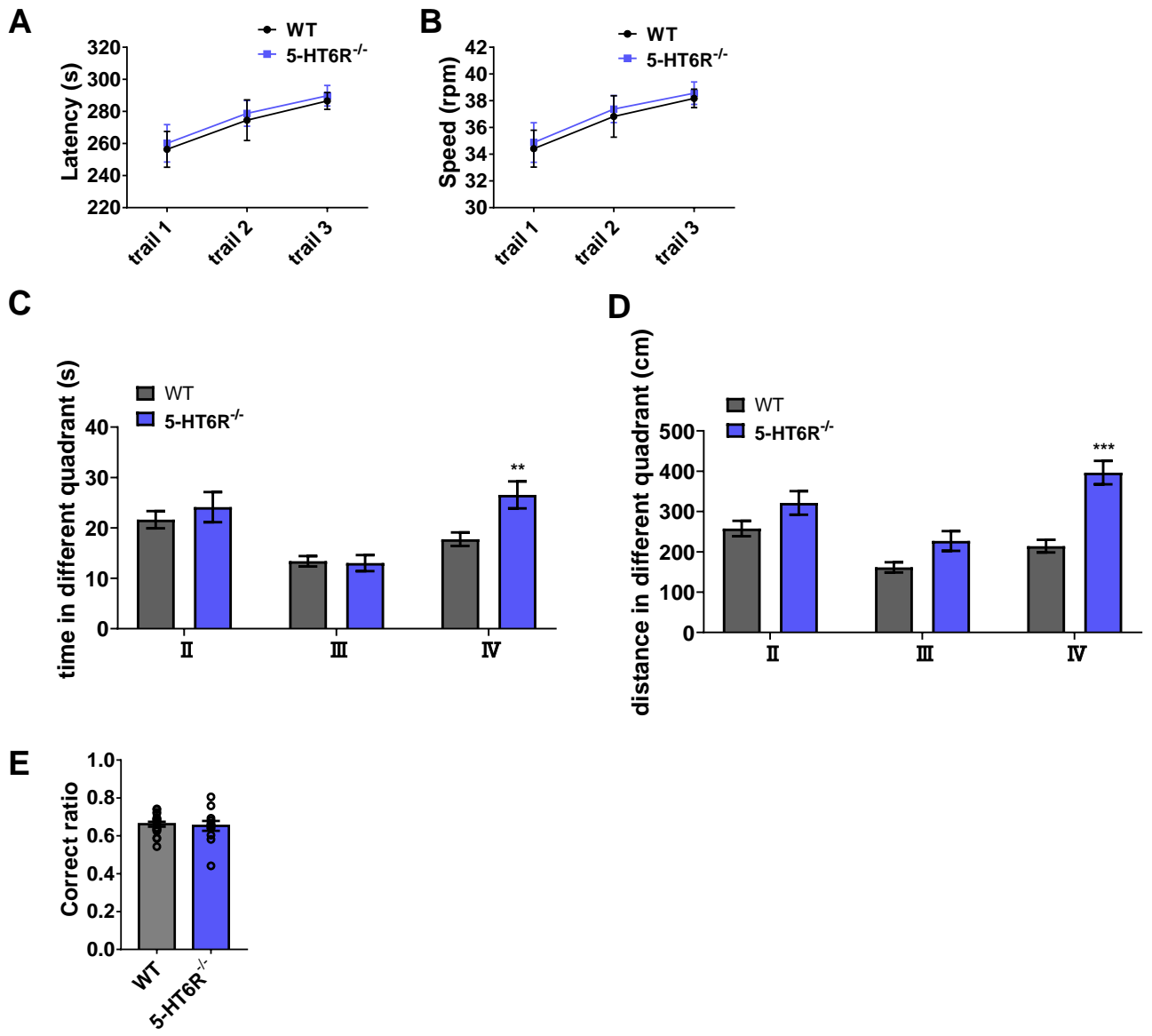

Fig.S3

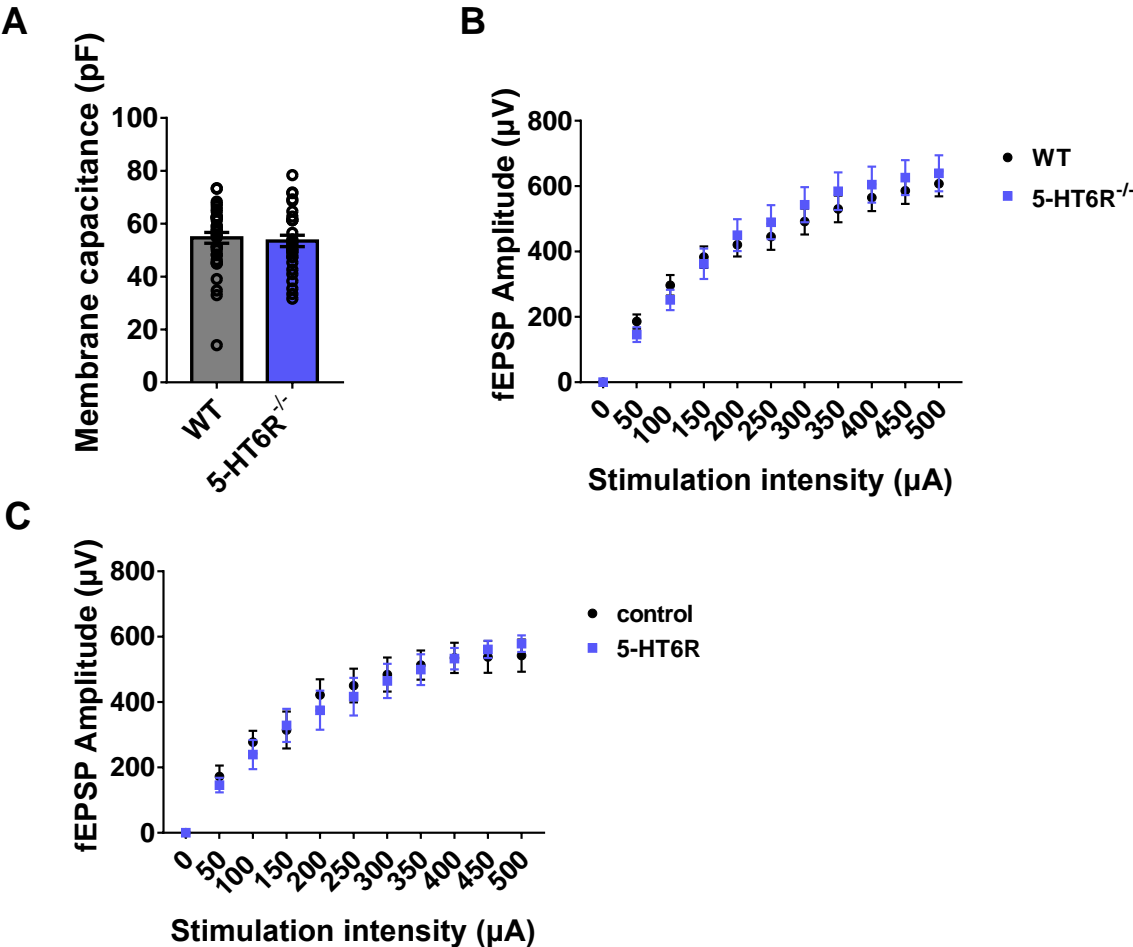

Fig.S4

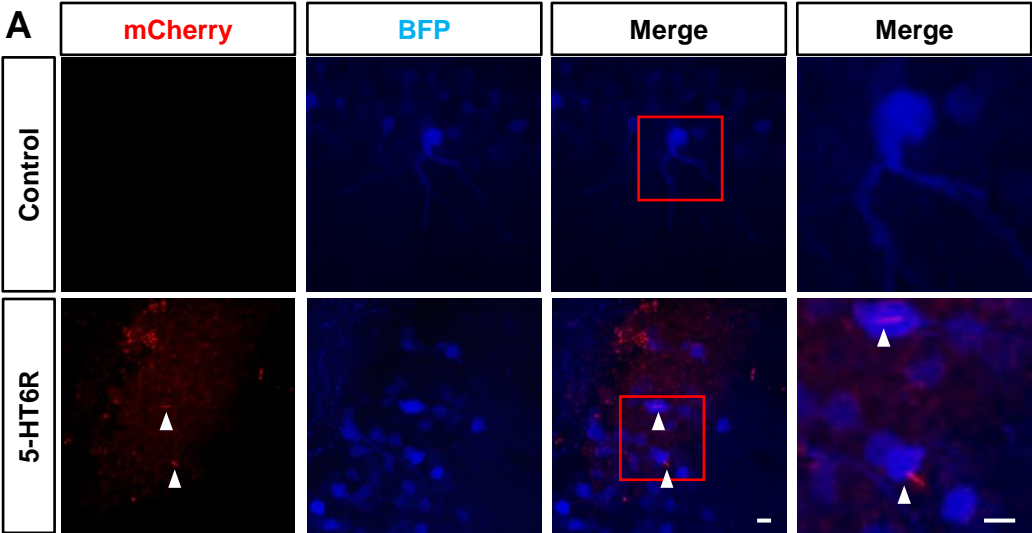

Fig.S5

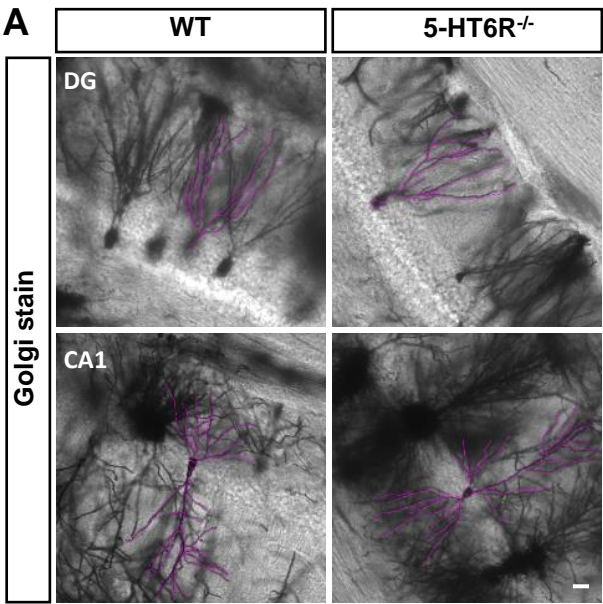

Supplement: Supplementary file 1 — Fig S1‐5 [file ACEL-20-e13369-s002.pdf]
